# Crystal structure of 6-chloro-5-(2-chloro­eth­yl)-3-(propan-2-yl­idene)indolin-2-one

**DOI:** 10.1107/S2056989015012268

**Published:** 2015-07-22

**Authors:** K. R. Roopashree, Gangadhar Y. Meti, Ravindra R. Kamble, H. C. Devarajegowda

**Affiliations:** a Department of Physics, Yuvaraja’s College (Constituent College), University of Mysore, Mysore 570 005, Karnataka, India; b Department of Studies in Chemistry, Karnataka University, Dharwad 580 003, Karnataka, India

**Keywords:** crystal structure, indolin-2-one, propan-2-yl­idene, hyaluronidase, disorder, N—H⋯O hydrogen bonding, inversion dimers

## Abstract

The title compound, C_13_H_13_Cl_2_NO, has a 3-(propan-2-yl­idene)indolin-2-one core with a Cl atom and a chloro­ethyl substituent attached to the aromatic ring. Two atoms of the aromatic ring and the chloro­ethyl substituent atoms are disordered over two sets of sites with a refined occupancy ratio of 0.826 (3):0.174 (3). In the crystal, mol­ecules are linked by pairs of N—H⋯O hydrogen bonds, forming inversion dimers with an *R*_2_^2^(8) ring motif.

## Related literature   

For inhibitors of hyaluronidase, see: Shen & Winter (1977 ▸). For the anti-inflammatory properties of some pyrido­pyrimidine derivatives, see: La Motta *et al.* (2007 ▸). For the synthesis and crystal structures of seven substituted 3-methyl­idene-1*H*-indol-2(3*H*)-one derivatives, including 3-(propan-2-yl­idene)indolin-2-one, see: Spencer *et al.* (2010 ▸).
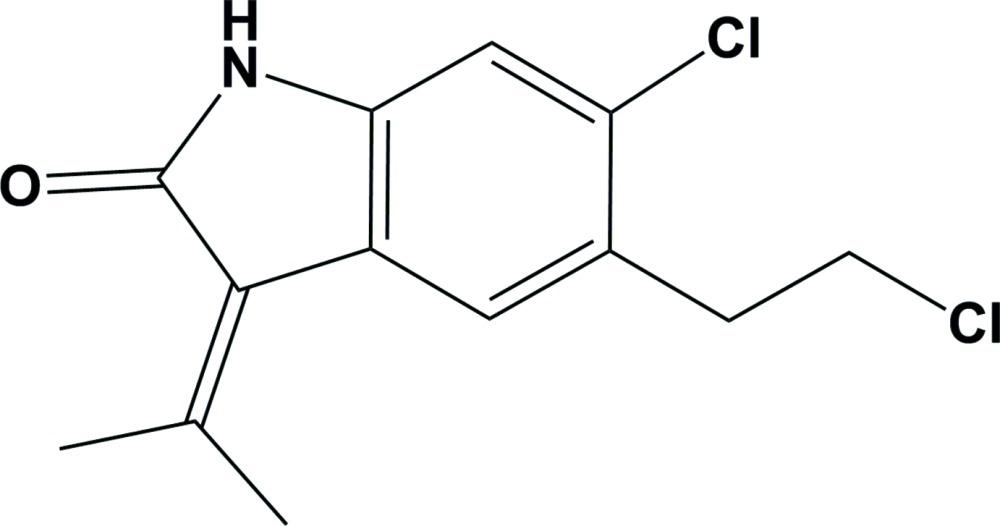


## Experimental   

### Crystal data  

C_13_H_13_Cl_2_NO
*M* *_r_* = 270.14
Triclinic, 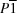

*a* = 8.1079 (10) Å
*b* = 8.8699 (10) Å
*c* = 9.1714 (12) Å
α = 101.136 (6)°
β = 97.799 (7)°
γ = 98.783 (6)°
*V* = 630.22 (14) Å^3^
*Z* = 2
Mo *K*α radiation
μ = 0.50 mm^−1^
*T* = 296 K
0.24 × 0.20 × 0.12 mm

### Data collection  

Bruker SMART CCD area-detector diffractometer
Absorption correction: multi-scan (*SADABS*; Bruker, 2001 ▸) *T*_min_ = 0.770, *T*_max_ = 1.000
25839 measured reflections
7740 independent reflections
4112 reflections with *I* > 2σ(*I*)
*R*_int_ = 0.026

### Refinement  

*R*[*F* ^2^ > 2σ(*F* ^2^)] = 0.045
*wR*(*F* ^2^) = 0.162
*S* = 1.01
7740 reflections
206 parameters
1 restraint
H atoms treated by a mixture of independent and constrained refinement
Δρ_max_ = 0.32 e Å^−3^
Δρ_min_ = −0.36 e Å^−3^

### 

Data collection: *SMART* (Bruker, 2001 ▸); cell refinement: *SAINT* (Bruker, 2001 ▸); data reduction: *SAINT*; program(s) used to solve structure: *SHELXS2014* (Sheldrick, 2008 ▸); program(s) used to refine structure: *SHELXL2014* (Sheldrick, 2015 ▸); molecular graphics: *PLATON* (Spek, 2009 ▸); software used to prepare material for publication: *SHELXL2014* and *PLATON* (Spek, 2009 ▸).

## Supplementary Material

Crystal structure: contains datablock(s) I, global. DOI: 10.1107/S2056989015012268/su5151sup1.cif

Structure factors: contains datablock(s) I. DOI: 10.1107/S2056989015012268/su5151Isup2.hkl

Supporting information file. DOI: 10.1107/S2056989015012268/su5151Isup3.cml

**Fig. 1.**
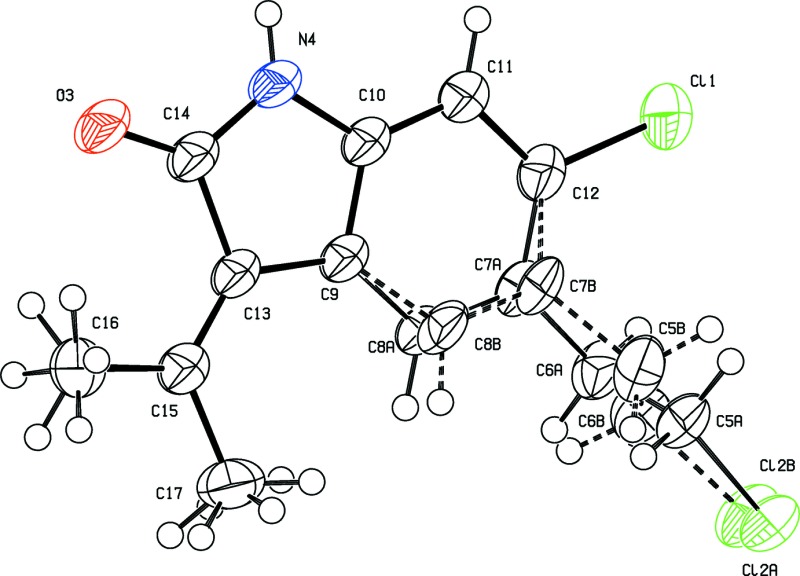
The molecular structure of the title compound, with atom labelling. Displacement ellipsoids are drawn at the 50% probability level. The dashed lines indicate the bonds involving the minor component atoms, C5B–C8B and Cl2B. The methyl groups have been treated as idealized-disordered with two positions rotated from each other by 60 °.

**Fig. 2.**
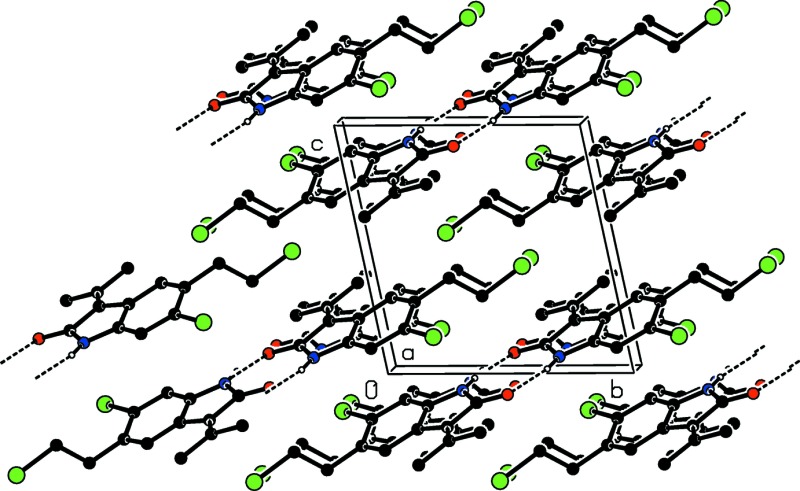
Crystal packing of the title compound, viewed along the a axis, with hydrogen bonds drawn as dashed lines (see Table 1 for details). The C-bound H atoms and the minor components of the disordered atoms have been omitted for clarity.

B B B . DOI: 10.1107/S2056989015012268/su5151fig1.tif
The mol­ecular structure of the title compound, with atom labelling. Displacement ellipsoids are drawn at the 50% probability level. The dashed lines indicate the bonds involving the minor component atoms, C5*B*–C8*B* and Cl2*B*. The methyl groups have been treated as idealized-disordered with two positions rotated from each other by 60 °.

a . DOI: 10.1107/S2056989015012268/su5151fig2.tif
Crystal packing of the title compound, viewed along the *a* axis, with hydrogen bonds drawn as dashed lines (see Table 1 for details). The C-bound H atoms and the minor components of the disordered atoms have been omitted for clarity.

CCDC reference: 1408952

Additional supporting information:  crystallographic information; 3D view; checkCIF report

## Figures and Tables

**Table 1:** Hydrogen-bond geometry (, )

*D*H*A*	*D*H	H*A*	*D* *A*	*D*H*A*
N4H4*N*O3^i^	0.84(1)	2.02(1)	2.8349(11)	166(2)

Symmetry code: (i) 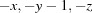.

## supplementary crystallographic information

### S1. Synthesis and crystallization

A mixture of 3-(2-chloro­ethyl)-2-methyl-4*H*-pyrido[1,2-a]pyrimidin-4-one (0.009 mol) and cyclic secondary amines (0.0108 mol) in the presence of *N*,*N*-diiso­propyl­ethyl­amine (0.015 mol) in aceto­nitrile (25 ml) were refluxed for 10–12 h. The reaction was monitored by TLC. After completion, the reaction mixture was filtered and washed with aceto­nitrile. Aceto­nitrile was then evaporated slowly giving the title compound as colourless plate-like crystals (yield: 70%; m.p.: 411–415 K).

### S2. Refinement

Crystal data, data collection and structure refinement details are summarized in Table 2. Atoms C7 and C8 of the aromatic ring and atoms C6—C5—Cl2 of the -CH_2_—CH_2_—Cl substituent are disordered over two positions (A and B) with a refined occupancy ratio of 0.826 (3):0.174 (3). The NH H atom was located in a difference Fourier map and refined with a distance restraint of 0.86 (2) Å. The C-bound H atoms were included in calculated positions and treated as riding atoms: C—H = 0.93 - 0.97 Å with U_iso_(H) = 1.5U_eq_(C) for methyl H atoms and 1.2U_eq_(C) for other H atoms. The methyl groups were treated as idealized-disordered (AFIX 123) with two positions rotated from each other by 60 °.

### S3. Structural commentary

Indomethacin (Indocin) and anti-allergic agents such as sodium cromoglycate and the natural product sorghumbran have been reported as inhibitors of hyaluronidase (Shen *et al.*, 1977). Some pyrido[1,2-*a*]pyrimidin-4-one derivatives have also been found to show anti-inflammatory properties (La Motta *et al.*, 2007). In an effort to develop a new class of non-steroidal anti-inflammatory drugs (NSAIDS) that inhibit hyaluronidase we have synthesized the title indoline derivative and report herein on its crystal structure.

The molecular structure of the title compound is shown in Fig. 1. Two atoms of the aromatic ring, C7 and C8, and the -CH_2_—CH_2_—Cl substituent (C6—C5—Cl2) are disordered over two positions (A and B) with a refined occupancy ratio of 0.826 (3):0.174 (3).

In the crystal, molecules are linked by a pair of N—H···O hydrogen bonds forming inversion dimers with an R^2^_2_(8) ring motif (Table 1 and Fig. 2).

### Crystal data

**Table:**

C_13_H_13_Cl_2_NO	*Z* = 2
*M**_r_* = 270.14	*F*(000) = 280
Triclinic, *P*1	*D*_x_ = 1.424 Mg m^−^^3^
*a* = 8.1079 (10) Å	Mo *K*α radiation, λ = 0.71073 Å
*b* = 8.8699 (10) Å	Cell parameters from 2227 reflections
*c* = 9.1714 (12) Å	θ = 2.3–25.0°
α = 101.136 (6)°	µ = 0.50 mm^−^^1^
β = 97.799 (7)°	*T* = 296 K
γ = 98.783 (6)°	Plate, colourless
*V* = 630.22 (14) Å^3^	0.24 × 0.20 × 0.12 mm

### Data collection

**Table:**

Bruker SMART CCD area-detector diffractometer	7740 independent reflections
Radiation source: fine-focus sealed tube	4112 reflections with *I* > 2σ(*I*)
Graphite monochromator	*R*_int_ = 0.026
ω and φ scans	θ_max_ = 40.6°, θ_min_ = 2.3°
Absorption correction: multi-scan (*SADABS*; Bruker, 2001)	*h* = −14→14
*T*_min_ = 0.770, *T*_max_ = 1.000	*k* = −16→15
25839 measured reflections	*l* = −16→16

### Refinement

**Table:**

Refinement on *F*^2^	Primary atom site location: structure-invariant direct methods
Least-squares matrix: full	Secondary atom site location: difference Fourier map
*R*[*F*^2^ > 2σ(*F*^2^)] = 0.045	Hydrogen site location: mixed
*wR*(*F*^2^) = 0.162	H atoms treated by a mixture of independent and constrained refinement
*S* = 1.01	*w* = 1/[σ^2^(*F*_o_^2^) + (0.0793*P*)^2^ + 0.0329*P*] where *P* = (*F*_o_^2^ + 2*F*_c_^2^)/3
7740 reflections	(Δ/σ)_max_ = 0.001
206 parameters	Δρ_max_ = 0.32 e Å^−^^3^
1 restraint	Δρ_min_ = −0.36 e Å^−^^3^

### Special details

**Table:**

Geometry. All esds (except the esd in the dihedral angle between two l.s. planes) are estimated using the full covariance matrix. The cell esds are taken into account individually in the estimation of esds in distances, angles and torsion angles; correlations between esds in cell parameters are only used when they are defined by crystal symmetry. An approximate (isotropic) treatment of cell esds is used for estimating esds involving l.s. planes.

### Fractional atomic coordinates and isotropic or equivalent isotropic displacement parameters (Å^2^)

**Table:**

	*x*	*y*	*z*	*U*_iso_*/*U*_eq_	Occ. (<1)
Cl1	0.45520 (4)	0.23428 (3)	0.16248 (5)	0.06608 (12)	
O3	−0.18925 (11)	−0.47480 (8)	0.07844 (11)	0.0570 (2)	
N4	0.03386 (12)	−0.28722 (9)	0.06869 (11)	0.0481 (2)	
H4N	0.0900 (19)	−0.3448 (17)	0.0209 (16)	0.060 (4)*	
C5A	0.14655 (19)	0.45717 (14)	0.31712 (16)	0.0492 (3)	0.826 (3)
H5A	0.0248	0.4222	0.2917	0.059*	0.826 (3)
H5B	0.1900	0.4621	0.2243	0.059*	0.826 (3)
C6A	0.22507 (18)	0.34151 (14)	0.39194 (15)	0.0474 (3)	0.826 (3)
H6A	0.3473	0.3735	0.4117	0.057*	0.826 (3)
H6B	0.1873	0.3420	0.4879	0.057*	0.826 (3)
C7A	0.1785 (2)	0.17773 (16)	0.2953 (2)	0.0415 (3)	0.826 (3)
C8A	0.0297 (2)	0.08170 (18)	0.3081 (2)	0.0413 (3)	0.826 (3)
H8A	−0.0388	0.1203	0.3744	0.050*	0.826 (3)
Cl2A	0.19269 (15)	0.64754 (8)	0.43853 (13)	0.0676 (2)	0.826 (3)
C5B	0.2356 (13)	0.4300 (10)	0.4328 (10)	0.071 (3)	0.174 (3)
H5C	0.3513	0.4122	0.4405	0.086*	0.174 (3)
H5D	0.1806	0.3749	0.5000	0.086*	0.174 (3)
C6B	0.1481 (12)	0.3659 (8)	0.2788 (10)	0.064 (2)	0.174 (3)
H6C	0.2072	0.4157	0.2105	0.076*	0.174 (3)
H6D	0.0344	0.3887	0.2691	0.076*	0.174 (3)
C7B	0.1384 (12)	0.1876 (7)	0.2341 (11)	0.0481 (17)	0.174 (3)
C8B	−0.0029 (14)	0.0948 (8)	0.2517 (10)	0.0476 (18)	0.174 (3)
H8B	−0.0919	0.1399	0.2833	0.057*	0.174 (3)
Cl2B	0.2382 (6)	0.6281 (5)	0.4909 (6)	0.0703 (10)	0.174 (3)
C9	−0.01760 (13)	−0.07171 (10)	0.22234 (11)	0.04093 (19)	
C10	0.09453 (12)	−0.12925 (9)	0.13136 (11)	0.04079 (19)	
C11	0.23847 (14)	−0.03796 (11)	0.11071 (13)	0.0458 (2)	
H11	0.3089	−0.0779	0.0471	0.055*	
C12	0.27408 (14)	0.11746 (11)	0.18961 (14)	0.0485 (2)	
C13	−0.16019 (13)	−0.19963 (10)	0.20926 (12)	0.04115 (19)	
C14	−0.11418 (13)	−0.33825 (10)	0.11333 (12)	0.0438 (2)	
C15	−0.30879 (13)	−0.20048 (10)	0.26028 (12)	0.0436 (2)	
C16	−0.44740 (16)	−0.33957 (14)	0.22317 (16)	0.0582 (3)	
H16A	−0.5159	−0.3334	0.3009	0.087*	0.5
H16B	−0.3996	−0.4328	0.2162	0.087*	0.5
H16C	−0.5161	−0.3423	0.1285	0.087*	0.5
H16D	−0.4385	−0.4056	0.1295	0.087*	0.5
H16E	−0.5548	−0.3062	0.2142	0.087*	0.5
H16F	−0.4383	−0.3967	0.3019	0.087*	0.5
C17	−0.34756 (17)	−0.05701 (15)	0.35607 (17)	0.0619 (3)	
H17A	−0.4680	−0.0661	0.3497	0.093*	0.5
H17B	−0.3035	0.0335	0.3207	0.093*	0.5
H17C	−0.2960	−0.0467	0.4590	0.093*	0.5
H17D	−0.2437	0.0132	0.4032	0.093*	0.5
H17E	−0.4082	−0.0864	0.4322	0.093*	0.5
H17F	−0.4156	−0.0062	0.2940	0.093*	0.5

### Atomic displacement parameters (Å^2^)

**Table:**

	*U*^11^	*U*^22^	*U*^33^	*U*^12^	*U*^13^	*U*^23^
Cl1	0.06298 (19)	0.04108 (14)	0.0925 (3)	−0.00484 (12)	0.02704 (16)	0.01226 (14)
O3	0.0557 (4)	0.0259 (3)	0.0826 (6)	0.0007 (3)	0.0216 (4)	−0.0057 (3)
N4	0.0511 (5)	0.0260 (3)	0.0636 (5)	0.0041 (3)	0.0207 (4)	−0.0043 (3)
C5A	0.0622 (8)	0.0274 (5)	0.0539 (7)	0.0057 (4)	0.0126 (6)	−0.0009 (4)
C6A	0.0556 (7)	0.0301 (5)	0.0482 (7)	−0.0009 (4)	0.0035 (5)	−0.0009 (4)
C7A	0.0486 (7)	0.0261 (5)	0.0457 (8)	0.0037 (4)	0.0028 (6)	0.0037 (5)
C8A	0.0477 (8)	0.0268 (5)	0.0470 (9)	0.0057 (4)	0.0113 (7)	0.0005 (6)
Cl2A	0.0900 (5)	0.02960 (18)	0.0798 (5)	0.0066 (2)	0.0337 (4)	−0.0064 (2)
C5B	0.085 (6)	0.049 (4)	0.069 (5)	−0.008 (4)	0.030 (4)	−0.008 (3)
C6B	0.087 (5)	0.036 (3)	0.073 (5)	0.009 (3)	0.023 (4)	0.017 (3)
C7B	0.061 (4)	0.0186 (19)	0.059 (5)	0.000 (2)	0.010 (4)	0.000 (3)
C8B	0.068 (5)	0.022 (2)	0.049 (4)	0.007 (2)	0.013 (4)	−0.001 (3)
Cl2B	0.083 (2)	0.0326 (11)	0.082 (2)	−0.0012 (10)	0.0189 (16)	−0.0141 (12)
C9	0.0450 (4)	0.0249 (3)	0.0507 (5)	0.0051 (3)	0.0128 (4)	0.0008 (3)
C10	0.0457 (5)	0.0260 (3)	0.0484 (5)	0.0051 (3)	0.0115 (4)	0.0013 (3)
C11	0.0499 (5)	0.0315 (4)	0.0555 (5)	0.0055 (3)	0.0170 (4)	0.0046 (4)
C12	0.0496 (5)	0.0303 (4)	0.0640 (6)	0.0014 (3)	0.0150 (4)	0.0075 (4)
C13	0.0454 (4)	0.0251 (3)	0.0507 (5)	0.0055 (3)	0.0118 (4)	0.0011 (3)
C14	0.0468 (5)	0.0265 (3)	0.0544 (5)	0.0046 (3)	0.0119 (4)	−0.0009 (3)
C15	0.0456 (5)	0.0323 (4)	0.0511 (5)	0.0058 (3)	0.0117 (4)	0.0036 (3)
C16	0.0528 (6)	0.0435 (5)	0.0740 (8)	−0.0027 (4)	0.0213 (5)	0.0046 (5)
C17	0.0553 (6)	0.0478 (6)	0.0780 (8)	0.0108 (5)	0.0236 (6)	−0.0069 (5)

### Geometric parameters (Å, º)

**Table:**

Cl1—C12	1.7396 (11)	C7B—C12	1.413 (8)
O3—C14	1.2297 (11)	C8B—C9	1.433 (7)
N4—C14	1.3609 (13)	C8B—H8B	0.9300
N4—C10	1.3919 (11)	C9—C10	1.4023 (13)
N4—H4N	0.836 (13)	C9—C13	1.4654 (13)
C5A—C6A	1.510 (2)	C10—C11	1.3727 (13)
C5A—Cl2A	1.7886 (14)	C11—C12	1.3942 (14)
C5A—H5A	0.9700	C11—H11	0.9300
C5A—H5B	0.9700	C13—C15	1.3500 (14)
C6A—C7A	1.5109 (19)	C13—C14	1.4954 (12)
C6A—H6A	0.9700	C15—C16	1.4871 (15)
C6A—H6B	0.9700	C15—C17	1.5017 (14)
C7A—C12	1.399 (2)	C16—H16A	0.9600
C7A—C8A	1.399 (2)	C16—H16B	0.9600
C8A—C9	1.4008 (18)	C16—H16C	0.9600
C8A—H8A	0.9300	C16—H16D	0.9600
C5B—C6B	1.455 (13)	C16—H16E	0.9600
C5B—Cl2B	1.731 (10)	C16—H16F	0.9600
C5B—H5C	0.9700	C17—H17A	0.9600
C5B—H5D	0.9700	C17—H17B	0.9600
C6B—C7B	1.542 (9)	C17—H17C	0.9600
C6B—H6C	0.9700	C17—H17D	0.9600
C6B—H6D	0.9700	C17—H17E	0.9600
C7B—C8B	1.353 (12)	C17—H17F	0.9600
			
C14—N4—C10	111.45 (8)	C7A—C12—Cl1	119.82 (9)
C14—N4—H4N	124.9 (11)	C7B—C12—Cl1	119.4 (3)
C10—N4—H4N	123.0 (11)	C15—C13—C9	130.75 (8)
C6A—C5A—Cl2A	111.04 (11)	C15—C13—C14	124.22 (9)
C6A—C5A—H5A	109.4	C9—C13—C14	104.91 (8)
Cl2A—C5A—H5A	109.4	O3—C14—N4	123.89 (9)
C6A—C5A—H5B	109.4	O3—C14—C13	129.12 (10)
Cl2A—C5A—H5B	109.4	N4—C14—C13	106.99 (8)
H5A—C5A—H5B	108.0	C13—C15—C16	123.31 (9)
C7A—C6A—C5A	111.94 (12)	C13—C15—C17	121.65 (9)
C7A—C6A—H6A	109.2	C16—C15—C17	115.01 (10)
C5A—C6A—H6A	109.2	C15—C16—H16A	109.5
C7A—C6A—H6B	109.2	C15—C16—H16B	109.5
C5A—C6A—H6B	109.2	H16A—C16—H16B	109.5
H6A—C6A—H6B	107.9	C15—C16—H16C	109.5
C12—C7A—C8A	117.68 (12)	H16A—C16—H16C	109.5
C12—C7A—C6A	123.44 (13)	H16B—C16—H16C	109.5
C8A—C7A—C6A	118.85 (14)	C15—C16—H16D	109.5
C7A—C8A—C9	120.94 (14)	H16A—C16—H16D	141.1
C7A—C8A—H8A	119.5	H16B—C16—H16D	56.3
C9—C8A—H8A	119.5	H16C—C16—H16D	56.3
C6B—C5B—Cl2B	112.9 (8)	C15—C16—H16E	109.5
C6B—C5B—H5C	109.0	H16A—C16—H16E	56.3
Cl2B—C5B—H5C	109.0	H16B—C16—H16E	141.1
C6B—C5B—H5D	109.0	H16C—C16—H16E	56.3
Cl2B—C5B—H5D	109.0	H16D—C16—H16E	109.5
H5C—C5B—H5D	107.8	C15—C16—H16F	109.5
C5B—C6B—C7B	111.6 (8)	H16A—C16—H16F	56.3
C5B—C6B—H6C	109.3	H16B—C16—H16F	56.3
C7B—C6B—H6C	109.3	H16C—C16—H16F	141.1
C5B—C6B—H6D	109.3	H16D—C16—H16F	109.5
C7B—C6B—H6D	109.3	H16E—C16—H16F	109.5
H6C—C6B—H6D	108.0	C15—C17—H17A	109.5
C8B—C7B—C12	118.7 (5)	C15—C17—H17B	109.5
C8B—C7B—C6B	117.4 (7)	H17A—C17—H17B	109.5
C12—C7B—C6B	123.6 (6)	C15—C17—H17C	109.5
C7B—C8B—C9	121.0 (7)	H17A—C17—H17C	109.5
C7B—C8B—H8B	119.5	H17B—C17—H17C	109.5
C9—C8B—H8B	119.5	C15—C17—H17D	109.5
C8A—C9—C10	117.85 (11)	H17A—C17—H17D	141.1
C10—C9—C8B	114.1 (4)	H17B—C17—H17D	56.3
C8A—C9—C13	134.61 (11)	H17C—C17—H17D	56.3
C10—C9—C13	107.42 (7)	C15—C17—H17E	109.5
C8B—C9—C13	132.8 (4)	H17A—C17—H17E	56.3
C11—C10—N4	127.85 (9)	H17B—C17—H17E	141.1
C11—C10—C9	123.12 (8)	H17C—C17—H17E	56.3
N4—C10—C9	109.03 (8)	H17D—C17—H17E	109.5
C10—C11—C12	116.87 (9)	C15—C17—H17F	109.5
C10—C11—H11	121.6	H17A—C17—H17F	56.3
C12—C11—H11	121.6	H17B—C17—H17F	56.3
C11—C12—C7A	122.91 (10)	H17C—C17—H17F	141.1
C11—C12—C7B	118.4 (3)	H17D—C17—H17F	109.5
C11—C12—Cl1	117.09 (8)	H17E—C17—H17F	109.5
			
Cl2A—C5A—C6A—C7A	176.40 (11)	C10—C11—C12—Cl1	−179.38 (8)
C5A—C6A—C7A—C12	90.18 (18)	C8A—C7A—C12—C11	−7.8 (2)
C5A—C6A—C7A—C8A	−87.90 (17)	C6A—C7A—C12—C11	174.13 (13)
C12—C7A—C8A—C9	2.7 (2)	C8A—C7A—C12—Cl1	177.11 (12)
C6A—C7A—C8A—C9	−179.14 (15)	C6A—C7A—C12—Cl1	−1.0 (2)
Cl2B—C5B—C6B—C7B	−176.5 (6)	C8B—C7B—C12—C11	24.6 (10)
C5B—C6B—C7B—C8B	94.2 (10)	C6B—C7B—C12—C11	−161.5 (7)
C5B—C6B—C7B—C12	−79.7 (11)	C8B—C7B—C12—Cl1	178.7 (6)
C12—C7B—C8B—C9	−1.6 (12)	C6B—C7B—C12—Cl1	−7.5 (11)
C6B—C7B—C8B—C9	−175.8 (8)	C8A—C9—C13—C15	12.5 (2)
C7A—C8A—C9—C10	4.3 (2)	C10—C9—C13—C15	−171.78 (11)
C7A—C8A—C9—C13	179.65 (13)	C8B—C9—C13—C15	−20.8 (5)
C7B—C8B—C9—C10	−19.6 (9)	C8A—C9—C13—C14	−171.37 (15)
C7B—C8B—C9—C13	−169.1 (6)	C10—C9—C13—C14	4.33 (11)
C14—N4—C10—C11	−179.28 (11)	C8B—C9—C13—C14	155.4 (5)
C14—N4—C10—C9	0.11 (13)	C10—N4—C14—O3	−176.79 (10)
C8A—C9—C10—C11	−6.94 (18)	C10—N4—C14—C13	2.67 (13)
C8B—C9—C10—C11	19.4 (5)	C15—C13—C14—O3	−8.40 (19)
C13—C9—C10—C11	176.52 (10)	C9—C13—C14—O3	175.16 (11)
C8A—C9—C10—N4	173.63 (12)	C15—C13—C14—N4	172.17 (11)
C8B—C9—C10—N4	−160.0 (5)	C9—C13—C14—N4	−4.27 (12)
C13—C9—C10—N4	−2.90 (12)	C9—C13—C15—C16	173.69 (11)
N4—C10—C11—C12	−178.46 (11)	C14—C13—C15—C16	−1.76 (18)
C9—C10—C11—C12	2.23 (16)	C9—C13—C15—C17	−4.40 (19)
C10—C11—C12—C7A	5.36 (18)	C14—C13—C15—C17	−179.85 (11)
C10—C11—C12—C7B	−24.8 (5)		

### Hydrogen-bond geometry (Å, º)

**Table:**

*D*—H···*A*	*D*—H	H···*A*	*D*···*A*	*D*—H···*A*
N4—H4*N*···O3^i^	0.84 (1)	2.02 (1)	2.8349 (11)	166 (2)

Symmetry code: (i) −*x*, −*y*−1, −*z*.
