# Supplementary figures and images for: Crystal structure of 6-chloro-5-(2-chloro­eth­yl)-3-(propan-2-yl­idene)indolin-2-one
Source: Acta Crystallogr E Crystallogr Commun. 2015 Jul 22;71(Pt 8):o592–3. doi: 10.1107/S2056989015012268 (PMC4571414; doi:10.1107/S2056989015012268)

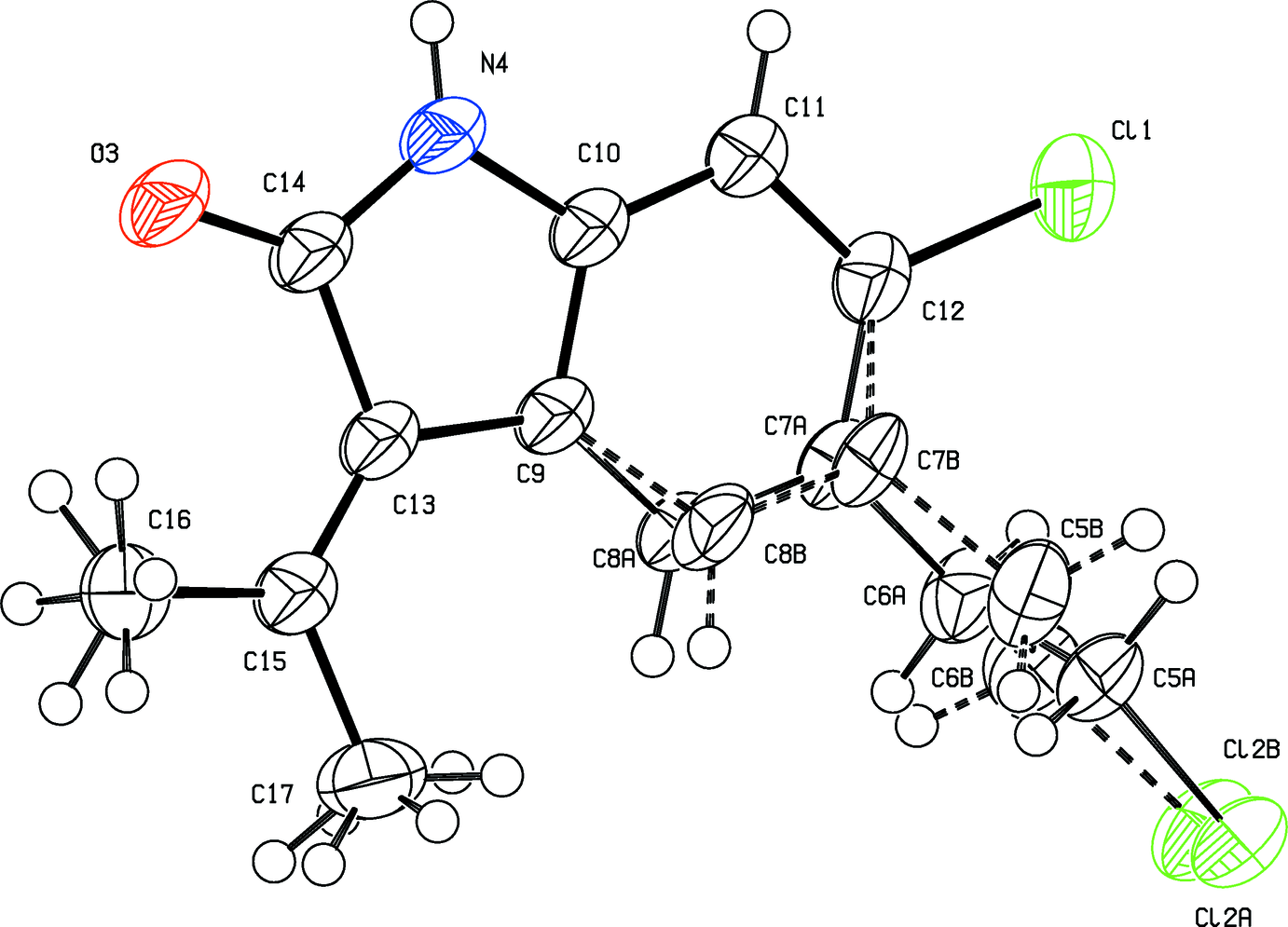

Supplement: Supplementary file 4 [file e-71-0o592-fig1.tif]

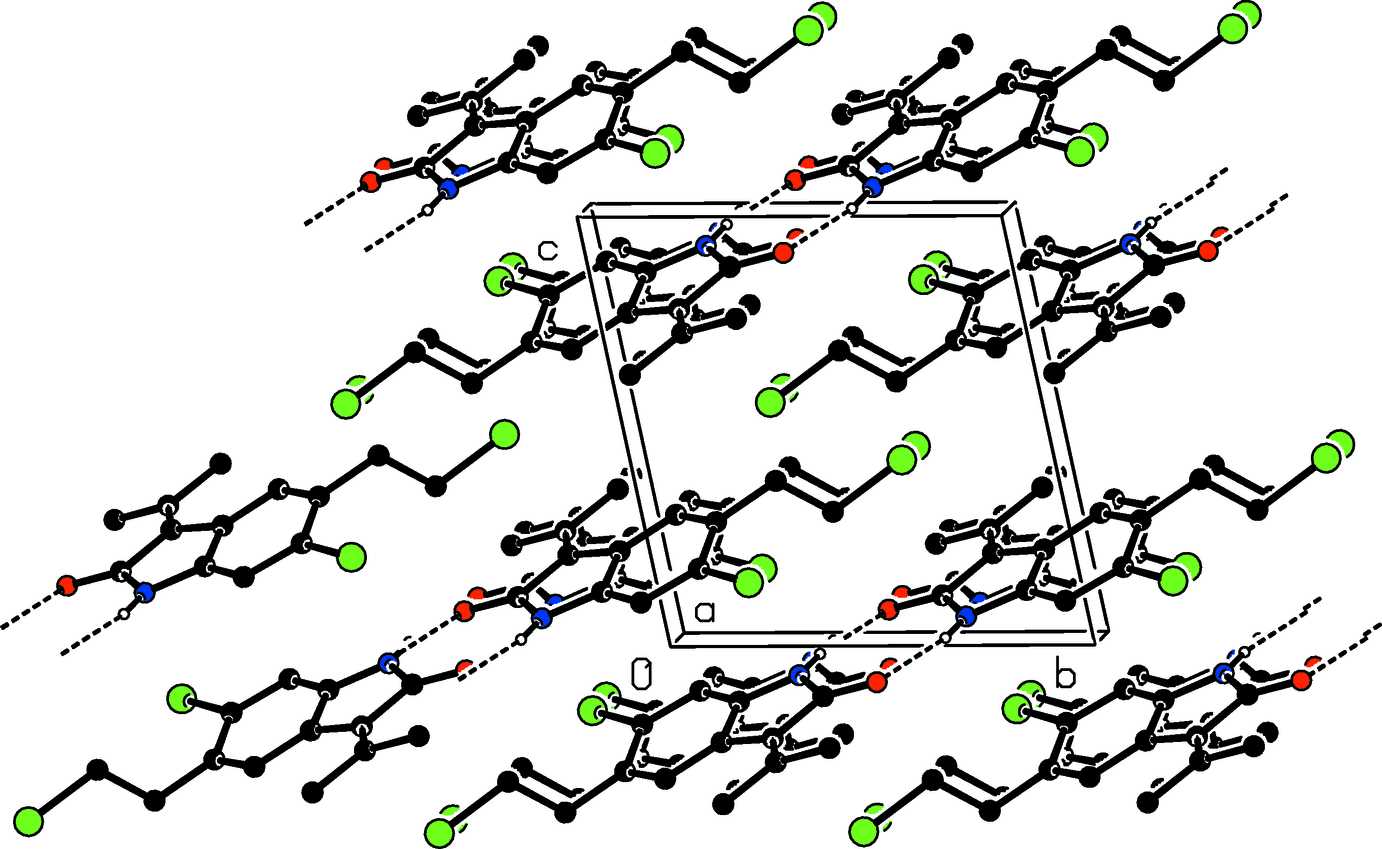

Supplement: Supplementary file 5 [file e-71-0o592-fig2.tif]
